# Calcined Mussel Shell Powder (CMSP) via Modification with Surfactants: Application for Antistatic Oil-Removal

**DOI:** 10.3390/ma11081410

**Published:** 2018-08-11

**Authors:** Danyi Wei, Hailong Zhang, Lu Cai, Jian Guo, Yaning Wang, Lili Ji, Wendong Song

**Affiliations:** 1 College of Marine and Electromechanical Engineering, Zhejiang Ocean University, Zhoushan 316022, China; wdy2dao4@163.com; 2 Institute of Innovation & Application, Zhejiang Ocean University, Zhoushan 316022, China; h_l_zhang@yahoo.com (H.Z.); wynzjou@126.com (Y.W.); 3 College of Environmental and Science Technology, Donghua University, Shanghai 201620, China; lucai89@126.com; 4 College of Food and Medical, Zhejiang Ocean University, Zhoushan 316022, China; gizsslt@126.com; 5 College of Petrochemical and Energy Engineering, Zhejiang Ocean University, Zhoushan 316022, China

**Keywords:** calcined mussel shell powder, modified, antistatic, oil cleaning, surfactant

## Abstract

Biomass is known to efficiently adsorb pollutants from wastewater. In this paper, we demonstrated that a new antistatic oil-cleaning material can be prepared and assembled by using two surfactants, alkyl polyglucosides (APG) and dimethyl octadecyl hydroxy ethyl ammonium nitrate (SN), to modify calcined mussel shell powder (CMSP) through a two-step hydrotherm-assisted adsorption. The pore size and structure of CMSP was measured by BET and a contact angle meter was used to characterize the surface wetting ability. XRD, FTIR, XPS, SEM, TEM, and HRTEM were employed to determine the surface structure of CMSP modified by surfactants APG and SN (MMO). In order to further characterize properties of the surface morphology and crystal structure, the HRTEM was employed to show that the MMO surface had a single crystal structure: calcite, with a crystal plane spacing of 0.2467 nm. The surface of MMO appeared to be fluffy and disperse. The antistatic and degreasing ability of as-prepared samples (MMO) was evaluated by a ZC-36 high resistance meter and BD-457 whiteness meter. The results showed that when the calcination temperature of CMSP reached 1000 °C, and the addition amount of APG and SN was 0.8 g and 0.16 g, it had an optimum antistatic effect with a surface resistivity (R*_s_*) of 1.35 × 10^8^ Ω, and a detergency rate to oil of 17.35%. This study aims to embrace a green solution to reduce environmental pressure and make use of waste, which is of great significance to environmental protection.

## 1. Introduction

Up to the present, natural bio-based materials derived from the low-cost, nontoxic, and renewable resources have attracted great interest to many material scientists [1,2,3,4]. There are two-fold benefits by exploring its application: firstly, to use these seemingly waste biomass materials to clean pollutants and, secondly, to recycle or make use of “waste materials” properly in order to minimize the risks of environmental pollution. There are some reports on the use of biomass materials to degrade oil pollution. Khan et al. [5] have carried out a bench-scale flume experiment to assess the oil removal and retention capabilities of biomass sorbents, and found that the sorbents that are hydrophobic, such as kapok fiber, cattail fiber, *Salvinia* sp., and polyester fiber, can remove more oil waste. Li et al. [6] have fabricated a versatile bio-based material (CH-PAA-T) via simple thermal cross-linking chitosan and polyacrylic acid for efficient water purification by removing insoluble oil, soluble toxic dyes, and heavy metal ions from water, simultaneously. Xu et al. [7] have prepared porous corncob and willow wood materials by the coagulation precipitation of biomass solution by water followed by washing and freeze-drying, which exhibited efficient adsorptive capacity towards methylene blue dye and oil. However, most of cleaning materials are focused on the preparation of superhydrophobic self-cleaning coatings and oil absorbing materials, which are not suitable for the petrochemical industry, which requires special attention to electrostatic hazards. Usually, at the base of oil storage, static electricity may also be generated due to friction, and static electricity may not be easily dissipated for non-metal tanks. The flow of oil in the pipelines and tanks can also generate electrostatic charge due to relative movements, such as flow, filtration, jetting, shock, and sedimentation [8]. All of this static electricity can cause serious accidents in oil tanks or in pipelines, leading to shocking damage and economic losses. Therefore, antistatic oil-cleaning materials will become one of the research directions for ensuring the safety of petrochemical enterprises. Many researchers are engaged in seeking novel antistatic agents, such as carbon nanotubes [9,10], intrinsically conductive polymers [11], and ionomers [12]. Zheng et al. [13] synthesized an antistatic PP (polypropylene) sheet sample, which is made of Tween and Span surfactants mixed with PP, and the best sample has a resistivity of 10^10^ Ω/sq. Unfortunately, these materials cannot clean oil. To our best knowledge, there is currently no other report on the use of biomass to prepare oil-removed antistatic materials. 

Mussel shell contains 5% organic matter, the rest is calcium carbonate [14,15,16], and this has become especially important due to its potential as a novel synthetic route to high-performance composite materials [17]. China is the most important world producer of the mussel [18], however, the use of shellfish is limited to edible parts. Currently, the feasibility of utilizing shell powders as adsorbents to remove dyes and metal ions from aqueous solutions has been investigated, which could benefit the environment [19]. Paradelo et al. [20] put forward that calcined mussel shell presented a higher retention capacity to P than the fine shell powder. Chowdhury et al. [21] discovered the maximum adsorption capacity of sea shell powder to Basic Green 4 was 42.33 mg/g at pH = 8. Hsu et al. [22] reported that oyster shell powder (OSP) can effectively adsorb Cu^2+^ and Ni^2+^ from wastewater with the adsorption capacities of the OSP toward Cu^2+^ and Ni^2+^ being 49.26–103.1 and 48.75–94.3 mg/g, respectively. Choi et al. [23] found that shell powder can enhance the shelf-life and the quality of kimchi for preservation and consumption. Peña-Rodríguez et al. [24] used batch and stirred flow chamber experiments to determine mercury retention on calcined and ground mussel shell, finding that calcined shell retained more Hg than ground shells (6300 vs. 4000–5200 μmol/kg). However, there are few reports on the preparation of functional materials by loading functional ions on the shell powder carrier. The rational use of biomass materials through self-assembly has been widely reported, the most widely used is cellulose, i.e., superhydrophobic matters [25], sensor matrix [26], photocatalysts [27], and antibacterial matters [27], which are based on its modified surface. 

Bio-based composite materials with specific surface modifications are hot topics in current research. How to design a bio-based composite material and utilize these biomass scaffolds is the direction of current research. In this work, a new antistatic oil-cleaning material for the oil-chemical industry was designed and prepared by using non-ionic surfactant alkyl polyglucosides (APG) and cationic surfactant dimethyl octadecyl hydroxy ethyl ammonium nitrate (SN) to modify calcined mussel shell powder (CMSP) through a two-step hydrothermal-assisted adsorption, which combines the functions of emulsifying oil and diverts net charge. The objective of this study is to evaluate the surface modification technology and make use of CMSP as a support scaffold material to construct an antistatic oil-cleaning shell-based material. It is of great significance to environmental protection to embrace a green solution to reduce environmental pressure and make use of waste. 

## 2. Materials and Methods 

### 2.1. Materials

Mussel shells (genus: *Mytilus* Linnaeus) were obtained from Shengsi, Zhoushan, China. Alkyl polyglucosides (APG, APG0810, 8–10 carbon alkyl chains, and its aqueous solution contained 50% alkyl polyglucosides) and dimethyl octadecyl hydroxy ethyl ammonium nitrate (SN, 50%) were purchased from Fine Chemical, Shanghai, China. A common detergent (Blue moon, Guangzhou, China) was purchased in the market. All chemical reagents were of analytical grade. All solutions were prepared by using deionized water. 

### 2.2. Preparation of CMSP

CMSP was prepared as follows: Mussel shells were firstly submerged into 1% HCl solution for 24 h at room temperature to remove surface impurities, and rinsed with deionized water until pH was neutral, then dried and crushed to a particle size less than 150 μm. The samples were loaded into a muffle furnace and heated to 300 °C, 500 °C, 800 °C, and 1000 °C for 2 h, respectively. Finally, calcined shells were smashed and finely ground by a micro-nano-pulverizer (MKCA6-2J, Masuko, Kawaguchi, Japan), and then sieved to <100 μm (average particle size: 75 μm) mesh particle size.

### 2.3. Modification of Shell Powder by Surfactants

CMSP modified by glucoside: 5 g CMSP was mixed with 0.08 g, 0.16 g, 0.4 g, 0.8 g, 1.6 g, and 2.4 g APG, respectively, and then each was dissolved in 35 mL deionized water with a high pressure reactor at 120 °C for 24 h. After filtration, the precipitate was dried at 60 °C overnight. The prepared samples were obtained and labeled as MMG. 

**Figure 1:**
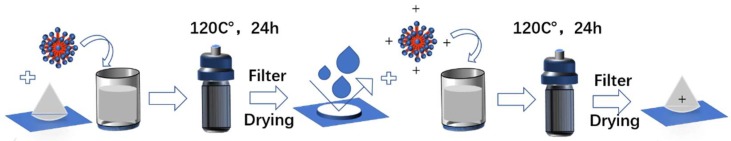
Main process steps for preparing antistatic, oil-cleaning, shell-based materials.

MMG modified by SN: 5 g MMG was mixed with 0.07 g, 0.1 g, 0.13 g, 0.16 g, and 0.32 g SN, respectively, and then each dissolved in 35 mL deionized water with a high pressure reactor at 120 °C for 24 h. After filtration, the precipitate was dried at 60 °C overnight. The prepared samples were obtained and labeled as MMO, and the main process steps are illustrated in Figure 1.

### 2.4. Research Method

The specific surface area and porosity tester (BET) was used to analyze the specific surface area and pore size distribution of shell powder under 77 K with N_2_ is the analysis gas (Quadrasorb SI, Quantachrome, Boynton Beach, LA, USA). The contact angle (CA) meter (XG-CAMB, Xuanyichuangxi Indstrial Equipment, Shanghai, China) was used to measured surface wetting ability of MMG, MMO by sessile-drop method [28] with a microsyringe at 20 °C. The injection volume of liquid is 5 mL and the average of nine readings of apparent contact angles is used as the final value for each sample [29]. 

The phase structures and crystallinity of CMSP, MMG, and MMO were characterized at room temperature by a powder X-ray diffractometer (XRD) at a setting of 40 kV and 30 mA (D8 ADVANCE Da Vinci, Bruker, Karlsruhe, Germany). ATR-FTIR (Nicolet 6700, Thermo Fisher Scientific, New York, NY, USA) was used to measure the functional groups and molecular structure of the as-prepared samples. For all spectra recorded, the specimens experienced a 64-scan data accumulation in the range of 600–4000 cm^−1^ at a spectral resolution of 4.0 cm^−1^. X-ray photoelectron spectra (XPS) were employed to measure chemical states of elements at the near surface of samples by using an X-ray photoelectron spectrometer (EscaLab 250Xi, Thermo Fisher Scientific, New York, NY, USA) based on a classic X-ray optic scheme in the range of binding energies from 0 to 1500 eV and the spot size is 500 μm. The grain shapes and morphologies of samples were investigated by scanning electron microscopy (SEM) (Quanta 200F, FEI, Hillsboro, OR, USA). Transmission electron microscopy (TEM) (F30, FEI, Hillsboro, OR, USA) and high-resolution transmission electron microscope (HRTEM) images were collected on a JEM-2100 (JEOL, Tokyo, Japan, with an acceleration voltage of 200 kV, dot resolution: 0.23 nm, line resolution: 0.14 nm, and magnification: 50×–1,500,000×). 

Surface resistance of MMO was measured using a ZC-36 high resistance meter (Shjingmi, Shanghai, China) at 23 °C, relative humidity 65% (65% RH), and 500 V, referring to China National Standard GB/T1410-1989, and duplicated three times. A whiteness meter (BD-457, Jingkelian Material Testing Machine, Tianjing, China) was used to measure the oil-cleaning capability of samples.

### 2.5. Determination of Oil Removal Rate 

Standard carbon black oil cloth (6 cm × 6 cm) and 1 g MMO were immersed in 150 mL hard water (0.304 g anhydrous CaCl_2_ and 0.139 g MgCl_2_ dissolved in 1000 mL deionized water), stirred in a magnetic stirrer at 120 rpm for 40 min and dried at 60 °C. At 25 °C, the decontamination ability of the material was measured by a white meter, and the measurements were repeated three times.

According to the whiteness value and the formula:

$$D_{r}=\frac{R_{w}-R_{s}}{R_{0}-R_{s}}\times 100\%$$

the sample wash rate can be calculated, where $R_{w}$ represents whiteness value after fouling, $R_{s}$ represents the whiteness value of the fouling cloth, and $R_{0}$ represents the whiteness value of the white cloth [30].

### 2.6. Statistical Analysis

Statistical analysis was performed using Origin 8.5 (Origin Corp., Hampton, MA, USA). Jade 6.0 was used to process the XRD data and analyze the major material composition of the sample. Digital Micrograph 3.9 was used to process TEM and HRTEM images, and to analyze the interplanar spacing and crystal type of the materials.

## 3. Results and Discussion

### 3.1. BET Measurement

**Figure 2:**
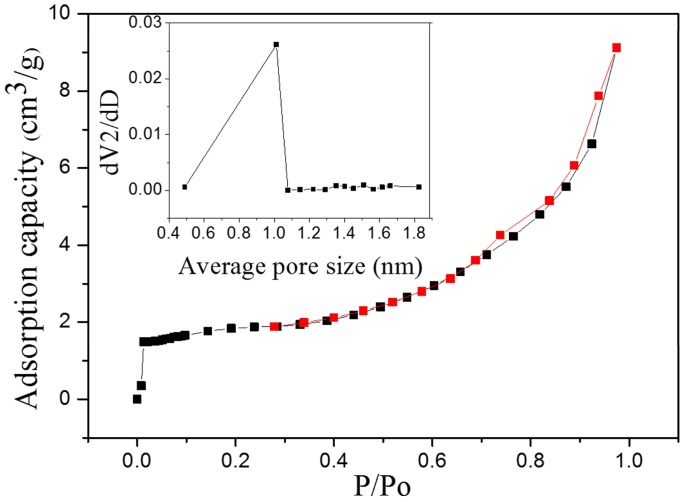
Isothermal adsorption curve and pore size distribution curve of CMSP.

According to IUPAC classification [31], we have found that the gas adsorption isotherm of CMSP is type IV, and the adsorption process is similar to macroporous adsorption. The adsorption capacity increases slowly due to the capillary condensation of a mesoporous solid after multi-layer adsorption. As the pressure increases, the capillaries condensed in the pores result in a sharp increase in adsorption [32]. In addition, as shown in Figure 2, it can be observed from the pore size distribution curve that the pore diameter of as-prepared samples is mostly between 0.5 nm and 1 nm, and the pore diameter is uniform and belongs to microporous material [31]. Compared with the untreated shell powder, the process of calcination can improve the pore structure of the shell powder, which is manifested as an increase in specific surface area and average pore size. The specific surface area increases from 1.120 m^2^/g to 6.004 m^2^/g, and the average pore size increases from 1.350 nm to 9.404 nm, as listed in Table 1.

### 3.2. Contact Angle Measurement 

**Figure 3:**
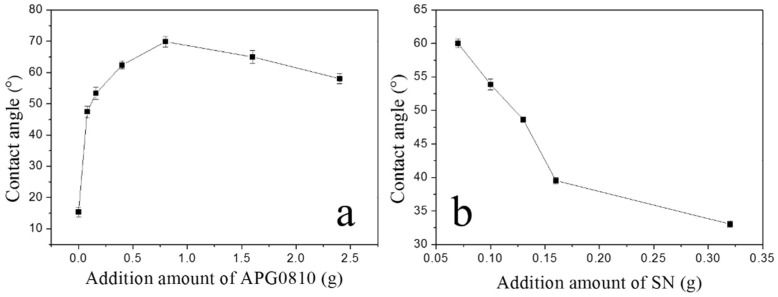
The apparent contact angle curves of MMG (**a**) and MMO (**b**) with different additional amounts of APG and SN.

The main component of mussel shell is calcium carbonate, and its polarity is high [33]. However, during the calcination process, calcium carbonate may become calcium oxide, and during the hydrothermal process, calcium oxide can be converted to calcium hydroxide. Here, both the hydrothermal process and the calcium hydroxide surface can promote the adsorption of surfactant APG through a polar interaction. According to the similarity intermiscibility theory, CMSP combines with the polar group hydroxyl of APG, and its hydrophobic end is exposed outside, causing the apparent contact angle of MMG to increase. As can be seen from Figure 3a, with the increase of the amount of APG, the apparent contact angle of MMG gradually increases and the apparent contact angle of the MMG becomes the largest when the additional amount is 0.8 g. When the additional amount is greater than 0.8 g, the apparent contact angle slowly decreases. The reason is that the surface of the calcined shell powder reaches the saturated state of adsorption, indicating the completion of APG monolayer adsorption. MMO is formed by the combination of MMG with the lipophilic group of cationic surfactants. Following the previous assumption, the surface should exhibit hydrophilicity. As illustrated in Figure 3b, with the increase of the amount of SN, the apparent contact angle of MMO gradually decreases, consistent with our hypothesis. 

### 3.3. XRD Characterization 

**Figure 4:**
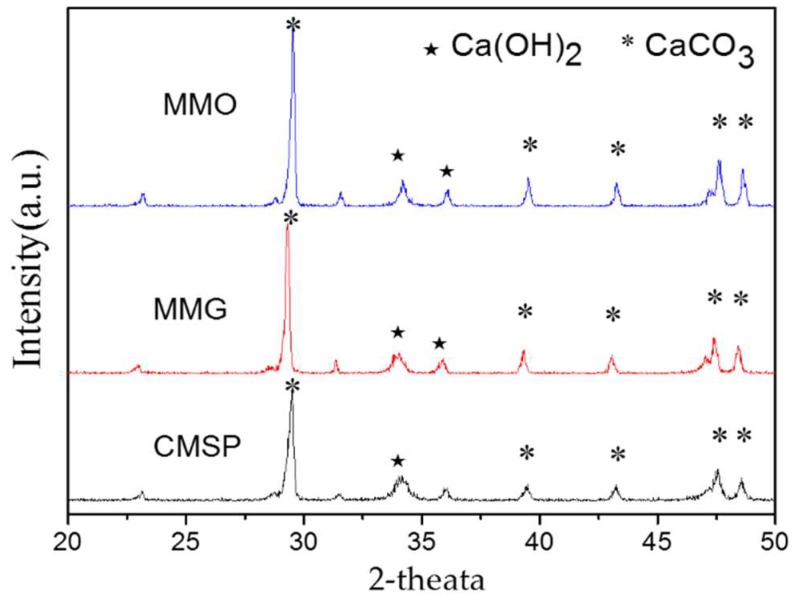
XRD patterns of CMSP, MMG, and MMO.

The crystalline structure of as-prepared samples is characterized by powder X-ray diffraction (XRD). Figure 4 presents the XRD patterns of CMSP, MMG, and MMO. Diffraction peaks at about 2θ = 29.52, 39.56, 43.27, 47.6, and 48.63 appear in the spectra, corresponding to (104), (113), (202), (018), and (116) crystal planes of CaCO_3_, all peaks can be indexed by calcite (JCPDS card No. 83-1762). The sharp, narrow diffraction peaks indicate that as-prepared calcium carbonate samples have a good crystallinity in all the morphologies of calcium carbonate [34]. In addition, there are two diffraction peaks at about 2θ = 34.068, 36.08 corresponding to Ca(OH)_2_. Since calcium carbonate is partially converted to calcium oxide after the calcination of CMSP at 1000 °C, and calcium oxide is converted into calcium hydroxide during the hydrothermal process. Since both surfactants are only single monolayers adsorbed on the surface of CMSP, they might not be detected by XRD. 

### 3.4. FTIR Analysis 

**Figure 5:**
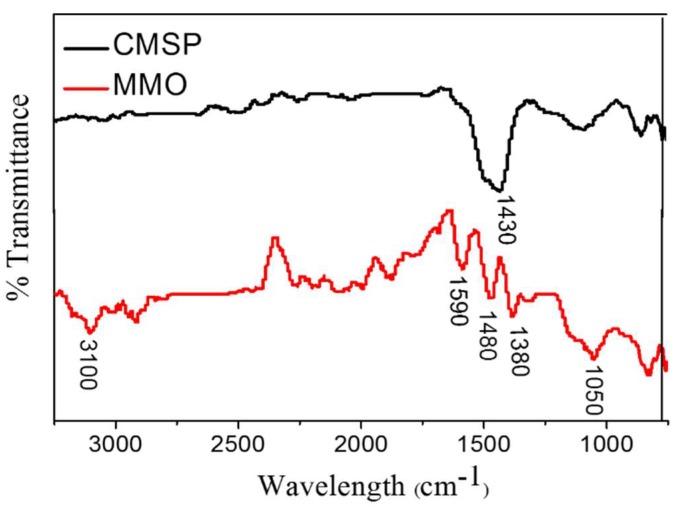
FTIR spectra of as-prepared sample CMSP and MMO.

FTIR spectra of CMSP and MMO are shown in Figure 5. Since the self-assembled layer of as-prepared samples is extremely thin, the intensity of infrared absorption peaks is weak. It can be observed that there is a peak at 1430 cm^−1^, anti-symmetrical stretching vibration of C-O in CO_3_^2−^, which is the fingerprint of CMSP. Absorption peaks were observed at 3100 cm^−1^ and 1050 cm^−1^ for sample MMO, which are O–H stretching vibration peaks and C–N stretching vibration peaks, respectively. Three absorption peaks at 1590 cm^−1^, 1480 cm^−1^, and 1380 cm^−1^ are assigned to the vibrational peak of benzene-like rings. The samples have been absolutely dried and there is no N–H stretching vibration in the quaternary ammonium salt, so the peak at 3100 cm^−1^ is considered to be the hydroxyl group of the self-assembled layer of MMO, and the peak at 1050 cm^−1^ is the C–N stretching vibration attributed to the quaternary ammonium salt of the MMO self-assembled layer. This can be the evidence that the surfactant molecule APG and SN are properly adsorbed on the CMSP surface. 

### 3.5. XPS Characterization

**Figure 6:**
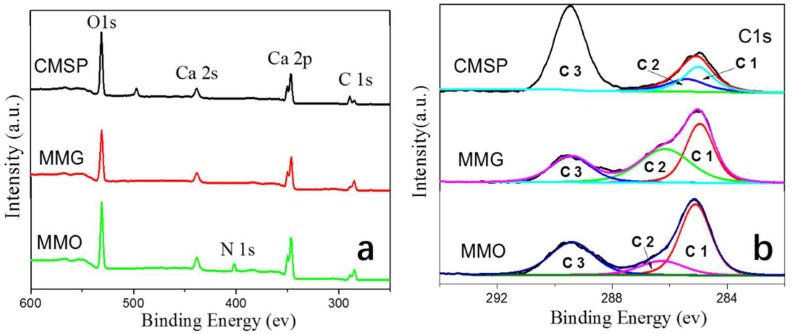
XPS spectra of CMSP, MMG, and MMO (**a**); and XPS survey scans (**b**).

In order to characterize the elements and content of the self-assembled layer on the shell powder surface, survey scans and high-resolution analysis of XPS on three as-prepared samples were carried out, and the results are shown in Figure 6. Figure 6a illustrated the survey scans of XPS spectrum of samples of CMSP, MMG, and MMO. By comparing the photoelectron peaks of the three samples, it was found that nitrogen appeared in the coating of MMO, with the binding energy of 402.2 eV, which indicated a coordination to three C atoms. As shown in Figure 6b, the peak area of carbon changed significantly. The C3 peak at 289.4 eV, representing MCO_3_ or CaCO_3_ in CMSP, corresponded to calcium carbonate in the shell powder. However, the intensity of the photoelectron peak of C3 calcium carbonate was significantly weakened for MMG and MMO after APG and SN self-assembling, respectively. Meanwhile the intensity of the XPS photoelectron peak at 285.2 eV increased suddenly, indicating that there was an organic layer covering the surface of samples MMG and MMO. This peak also became broad and wide, which indicated it contained at least two peaks, C1 (285.0 eV) and C2 (286.4 eV), attributed to organic carbon C–C and C–O bonds, respectively. These XPS and ATR-FTIR results suggested that there were organic layers successfully loaded on the surface of the shell powder. Furthermore, the nitrogen signal (N) only appeared at the surface of the MMO sample, which illustrated that a self-assembled mono-layer SN loads on the surface of MMO.

### 3.6. SEM Characterization 

**Figure 7:**
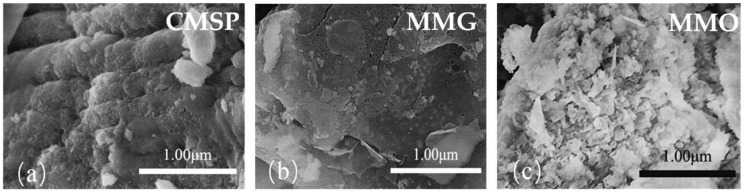
SEM images of CMSP (**a**); MMG (**b**); and MMO (**c**) at a magnification of 50,000×.

SEM photographs of as-prepared samples CMSP, MMG, and MMO are shown in Figure 7. It can be seen from Figure 7a that CMSP has a very small regular layer structure, and MMG has a smooth compacted surface in Figure 7b and, in Figure 7c, MMO appears as flake-shaped fragments on the surface. The structure of MMG and MMO matches the previous experimental hypothesis that MMG was composed of CaCO_3_, Ca(OH)_2_, and adsorbed via the hydroxy groups of APG, showing the properties of hydrophobic groups with a smooth surface. MMO was formed by adsorption of surfactant SN via its hydrophobic groups on the surface of MMG, leaving its lipophilic group outwards. As a result, the surface of the as-prepared MMO samples exhibited highly porous and dispersive characteristics. 

### 3.7. TEM Characterization

**Figure 8:**
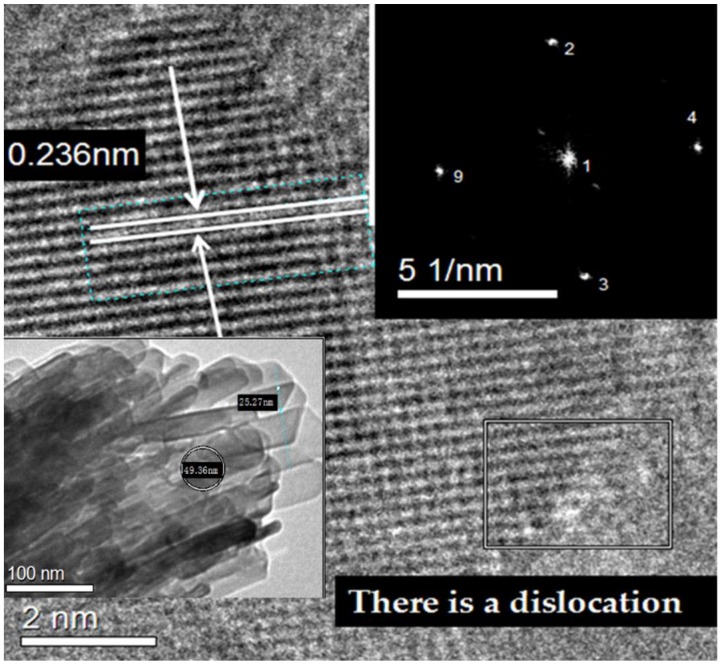
TEM and HRTEM images of MMO.

As illustrated from the TEM image of MMO (Figure 8), two forms of morphological substances can be found: stick-shaped and elliptical. However, it can be seen from the crystallographic diffraction fringes of HRTEM that MMO only has one crystal structure and the crystal plane spacing is about 0.2467 nm: the crystal structure is calcite corresponding to (110) planes, after analysis and calculation, consistent with the XRD results. However, dislocations are present in the crystal structure, which may be a result of the arrangement of extra atomic layers. Whether this is related to the self-assembling layer of surfactants remains to be further investigated. 

Video S1 is a clip of MMO photos taken at different point in time under a transmission electron microscope. The process of gradual decomposition of organic matter on the surface of MMO under the electron beam over time can be seen from this video. Please refer to the section of “Supplementary Materials” for details.

### 3.8. Surface Resistance of MMO 

**Figure 9:**
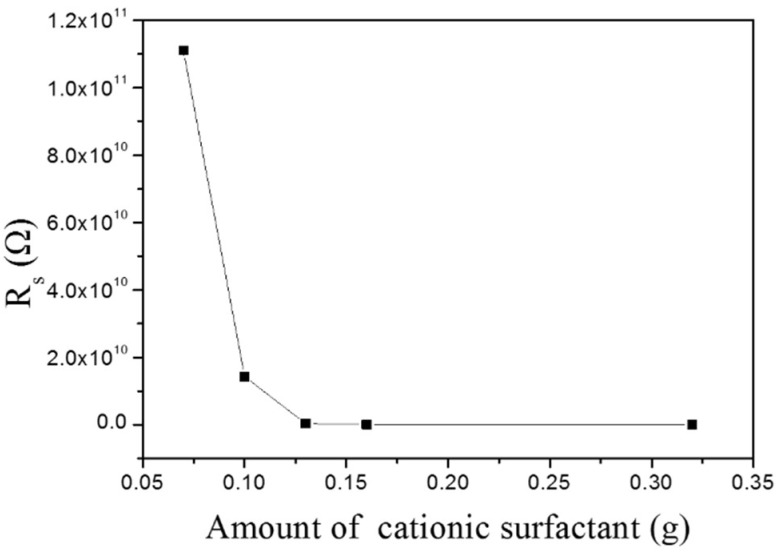
R*_s_* of MMO with different additional amounts of SN (65% RH, 500 V).

The MMG surface can be adsorbed by quaternary ammonium salt cationic surfactant: hydrophobic groups presented on the surface of MMG interact strongly with hydrophobic groups of SN through intermolecular interactions, and then, the positively-charged hydrophilic end (-N^+^(C_2_H_6_)) is exposed. Therefore, the surface of MMO is not only hydrophilic, but also has a positive charge. It can be seen from Figure 9 that the surface resistance of the sample gradually decreases with the increase of the additional amount of SN. When the additional amount of the modifier exceeds 0.16 g, the change of the surface resistance appears to be gentle, $R_{s}$ is 1.35 × 10^8^ Ω (specific surface area is 3.0434 per square inch), which has reached the antistatic level. The surface resistivity of the static dissipative material is 10^6^–10^9^ ohms per square inch based on GB 12158-1990 “General Guidelines for Anti-Static Incidents” and GJB 3007-1997 “Anti-Static Workspace Technical Requirements” [35].

### 3.9. Detergency Rate of MMO 

**Figure 10:**
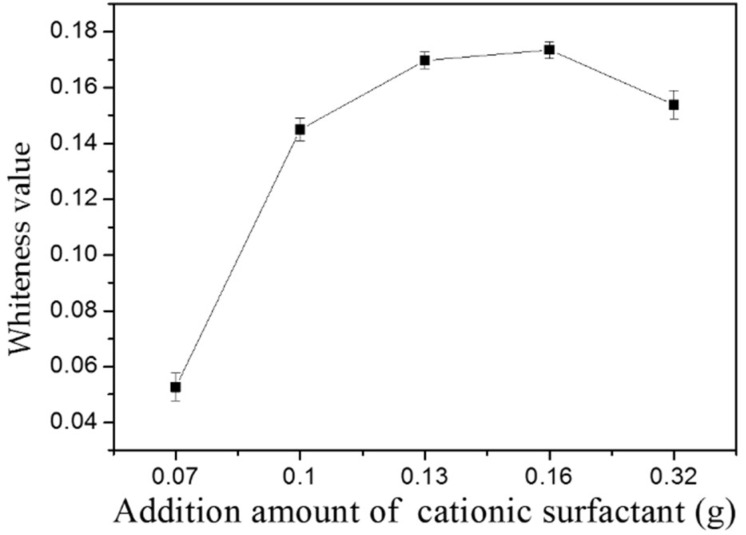
The detergency rate of MMO with different additional amounts of SN.

It can be seen from Figure 10 that the detergency rate of MMO increases as the concentration of cationic surfactant SN increases. When the additional amount of SN is 0.16 g, the detergency rate of MMO reaches its maximum, i.e., 17.35%. However the combination of SN and APG leads to the growth of the carbon chain on the surface of sample. As a result, the CMC value of this whole hybrid system has decreased [36], and then the surface tension would be decreased, finally, the decontamination rate of the sample has been decreased. As a comparison, a common brand detergent in the market has been selected and tested: its detergency rate can reach 18.77%, without antistatic effect. Similar to the washing mechanism of the surfactant, the carbon black oiled cloth is first wetted by hard water, then MMO wets the cloth and make the oil gradually curl into oil beads and, finally, with rinsing, flushes away from the surface of the cloth [37]; the shell powder also has an adsorption effect on oil stains.

Table 2 shows a comparison of the degreasing effect of several sorbents found in the literature. There have been many reports on oil-adsorbing materials. Most of them use a material with high specific surface area and super-lipophilicity to construct an adsorbent material [38,39,40]. This kind of material can greatly improve the treatment of oily wastewater, but it may not apply to petrochemical companies that require special attention to electrostatic hazards. Based on the emulsification mechanism, the as-prepared sample MMO can directly emulsify oil and, at the same time, static charge generated on the surface, e.g., during a wiping process, can be eliminated because MMO itself has a positive charge. As shown in Table 2, the oil-removal ability of three surfactants [41,42,43] and MMO is compared. It has been found that the oil removal rate of MMO is almost the same as that of the first oil-specific surfactant. The cleaning index of SDS/IC_13_ EO_6_ (2:1) is 20.27% [41], and the oil removal rate of MMO is 17.35%. The main components of standard carbon black oil cloth JB-02 are acetone (insoluble matter), phospholipids, carbon black powder, and gum arabic. Due to its complex oily composition, it usually needs to be aged for one week after preparation, as the dirt is more difficult to remove than artificial soil. By comparison, MMO not only achieves a similar cleaning rate as an oily detergent, but also has its antistatic properties that are not found in other detergents.

Nanostructures with specific surface modifications are the direction of current research, especially for natural bio-based materials. How to design a bio-based composite material and make use of these biomass scaffold materials is a hot topic in nanoscience and bioscience today. Self-assembled monolayers are well studied on the smooth and ideal surface. To the best of our knowledge there is no report about SAMs on porous calcinated shell powder surfaces. In this work, biodegradable polymer/surfactants can be directly assembled onto the porous calcinated shell powder surface. Through a two-step hydrotherm assisted process, two surfactants, non-ionic APG and cationic SN, are used, for the first time, to adsorb and modify the surface of porous CMSP with an average pore size 9.4 nm. The analysis results of both FTIR and XPS show two surfactants adsorb successfully on the porous surface of CMSP. 

**Figure 11:**
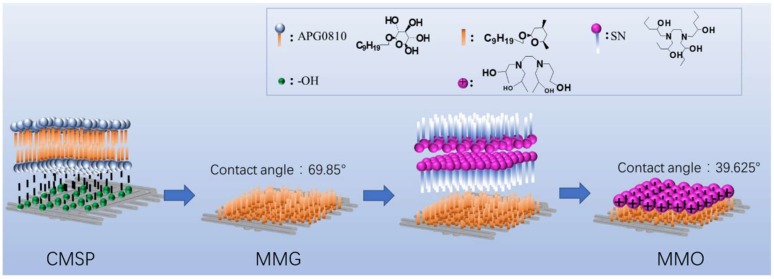
Schematic diagram of surfactant molecules APG and SN interacting with the surface CMSP during the two-step hydrotherm-assisted adsorption.

As shown from Figure 11, CMSP has a polar -OH group on the surface after the hydrothermal process, through the polar interaction with a non-ionic surfactant molecule, glucoside (APG). APG molecules physically adsorb on the CMSP surface via the hydrophilic group (-OH) of APG to form a self-assembled mono-layer on the surface of MMG. In the following adsorption step, the surface of MMG is combined with the lipophilic group of the cationic surfactant (SN) through hydrophobic interaction to form a self-assembled monolayer on the surface of MMO, with N^+^ ions attached to the surface of the MMO, which showed the positive electrical charge and with modest hydrophilic character.

In this work, CMSP decorated with surfactants via a hydrothermal-assisted method has been tested and shows its capability of both oil-cleaning and antistatic properties, and this bio-based material can be designed to gain a low-cost, environmentally friendly cleaning material, which can be used in petrochemical, aerospace, and other fields due to its unique antistatic properties. 

## 4. Conclusions

In summary, we have demonstrated that CMSP decorated with surfactants via a hydrothermal-assisted adsorption could significantly improve both its oil-cleaning and antistatic properties. The surface morphology of the as-prepared sample is highly dispersive, compared with CMSP and MMG, and it can be seen from SEM images that the surface morphology of the shell powder has undergone a real change through the self-assembly process. Additionally, HRTEM indicates that its crystal plane spacing is 0.236 nm, with a single crystal structure of calcite. The antistatic and degreasing ability of as-prepared samples has been assessed that the detergency rate to oil can reach as high as 17.35%, and also reach an antistatic level: the surface resistance is 1.35 × 10^8^ Ω. Biodegradable polymer/surfactants have been successfully assembled for the first time on to the surface of calcined shell powder. Therefore, the surface properties of CMSP are modified via combining two surfactants, non-ionic APG and cationic SN, to gain a low-cost, environmentally friendly cleaning material.

Compared with the existing antistatic materials, our prepared samples can clean oil with a low cost and, due to these characteristics, it can be industrialized and used in petrochemical, aerospace, and other fields, with broad application prospects. The washing durability of this material requires further study.

## Figures and Tables

**Table 1:** Specific surface area and average pore size of as-prepared samples at different puffing temperature.

Temperature (°C)	Specific Surface Area (m^2^/g)	Average Pore Size (nm)
blank	1.120	1.350
300	2.516	3.826
500	1.308	3.330
800	5.539	10.615
1000	6.004	9.404

**Table 2:** The comparison of the degreasing effect of several sorbents found in the literature.

Degreasing Mechanism	Sorbent	Degreasing Effect	Action Object	Ref.
Adsorption	Polymethylmethacrylate	55 g/g.	motor oil	[38]
	(ChiFer(III))	0.0138 g/g	Neodymium	[39]
	N-doped reduced graphene oxide aerogel	210 g/g.	Crude oil	[40]
Emulsification	(SDS) + isomeric alcohol ethoxylate (IC1_3_EO_6_)	20.27%	Standard carbon black oil cloth JB-02	[41]
	LP(A)	95.00%.	The artificial soil	[42]
	SA08-07(C_28_H_53_O_13_)	91.60%	The artificial soil	[43]
	MMO	17.35%	Standard carbon black oil cloth JB-02	In this work

## Supplementary Materials

The following are available online at http://www.mdpi.com/1996-1944/11/8/1410/s1, Video S1: Change of organic layer on the material’s surface.
